# SARS-CoV-2 Infection Inducing Immune Thrombocytopenic Purpura: Case Series

**DOI:** 10.31486/toj.20.0100

**Published:** 2021

**Authors:** Nancy Guirguis, Tyler Rehman, Yara Shams, Melanie Gordon, Essam Mekhaiel, Shaina Rozell, Mauna Pandya

**Affiliations:** ^1^ Advocate Christ Medical Center Internal Medicine Residency, University of Illinois at Chicago, Oak Lawn, IL; ^2^ Department of Internal Medicine, Advocate Christ Medical Center, Oak Lawn, IL; ^3^ Department of Pulmonology and Critical Care, Advocate Christ Medical Center, Oak Lawn, IL; ^4^ Department of Hematology and Oncology, Advocate Christ Medical Center, Oak Lawn, IL

**Keywords:** *COVID-19*, *immune thrombocytopenic purpura*, *SARS-CoV-2*

## Abstract

**Background:** Immune thrombocytopenic purpura (ITP) refers to immune-mediated destruction of platelets. Viral infections have been proposed as an etiology of ITP; antibodies developed in response to infection may cross-react with normal platelets and result in their destruction.

**Case Series:** We report 2 cases in which coronavirus disease 2019 (COVID-19) likely induced severe ITP.

**Conclusion:** ITP may also play a role in the thrombocytopenia observed in some patients with COVID-19. ITP in this patient population may be more prevalent than currently documented.

## INTRODUCTION

Immune thrombocytopenic purpura (ITP) refers to immune-mediated destruction of platelets, which can be associated with increased risk for bleeding. While the pathogenesis remains unclear, platelet-reactive antibodies may be triggered by environmental factors such as viral infections or concomitant autoimmune disorders.^1^ An immune response against viral infections results in the activation of cytotoxic T cells that results in a cascade of thrombopoiesis and eventual apoptosis of platelets.^2^ The relationship between coronavirus disease 2019 (COVID-19) and ITP has not yet been established. Similar to the pathogenesis of viral-induced ITP, we propose that severe acute respiratory syndrome coronavirus 2 (SARS-CoV-2) viral antigens, developed in response to the infection, cross-react with normal platelets and subsequently destroy or prevent their release.^2,3^ We report 2 cases in which SARS-CoV-2 infection likely induced severe ITP.

## CASE SERIES

### Case 1

**Figure 1.**
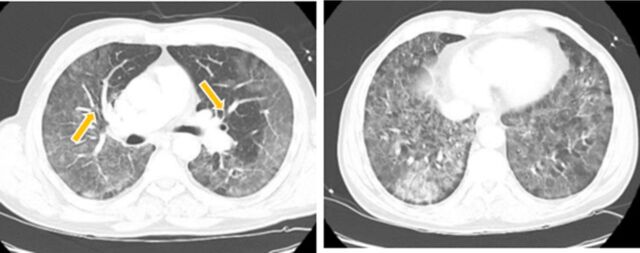
Computed tomography images show filling defect (arrows, left panel) suggestive of pulmonary emboli and extensive ground-glass opacities (right panel) in bilateral lungs.

A 63-year-old male with no significant medical history presented with cough, fever, and shortness of breath that began 1 week prior to arrival. Imaging revealed extensive bilateral peripheral infiltrates, suggestive of SARS-CoV-2 infection, as well as bilateral distal pulmonary emboli (Figure 1). He was admitted for acute hypoxic respiratory failure secondary to COVID-19 pneumonia and bilateral distal pulmonary emboli. Initial laboratory results were significant for lymphopenia; normal platelet count of 264,000 μL; and elevated ferritin, lactate dehydrogenase, C-reactive protein, and D-dimer.

On day 6 of admission, the patient's platelet count decreased more than 50% to 102,000 μL. He had had no exposure to heparin, and platelet factor 4 testing was negative, arguing against heparin-induced thrombocytopenia. His platelet count continued to decrease significantly, with a nadir count of 15,000 μL. No other obvious etiologies of thrombocytopenia were identified. Peripheral blood smear revealed giant platelets, raising suspicion for ITP. Other differentials included consumptive platelet loss in the setting of shock, acute liver injury, and bone marrow suppression. The patient remained hemodynamically stable, however, without any evidence of shock or liver disease. Additionally, because the patient had no other cytopenias, bone marrow suppression was also unlikely.

The patient was diagnosed with SARS-CoV-2–induced ITP and treated with 2 doses of 1 mg/kg intravenous immunoglobulin (IVIG). After the initial IVIG dose, the patient's platelet count increased to 45,000 μL. After the second dose of IVIG, the patient's platelet count normalized to 197,000 μL and remained normal for the duration of his hospitalization.

### Case 2

**Figure 2.**
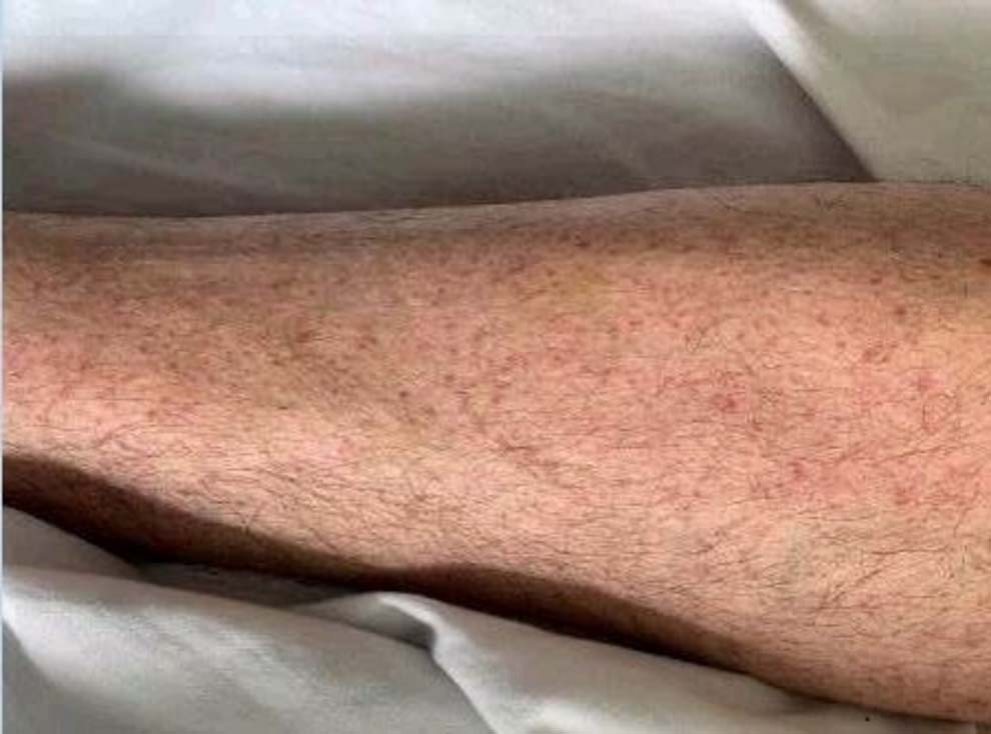
Rash on the left leg (Case 2) demonstrates nonblanching diffuse petechiae.

A 57-year-old male presented with a diffuse petechial rash of 3 days’ duration (Figure 2). His medical history included hypertension treated with lisinopril. He reported malaise, fever, and chills 3 weeks prior to arrival that had since resolved. In the emergency department, initial laboratory results showed a platelet count of 4,000 μL with no other concerning complete blood count abnormalities. Chest radiograph showed minimal perihilar interstitial opacities without focal lobar consolidation, suggestive of atypical pneumonia, pulmonary hemorrhage, or pulmonary edema. The patient had no hemoptysis, leukocytosis, or other symptoms to suggest acute heart failure.

Because of his viral prodrome and nonspecific chest radiograph, the patient was screened for SARS-CoV-2 infection and diagnosed with COVID-19 pneumonia. He did not have any respiratory or neurologic complaints and did not require any oxygen supplementation throughout his hospital stay. He had a normal haptoglobin level and an unremarkable peripheral blood smear.

The patient was treated with 2 doses of 1 mg/kg IVIG and 3 doses of 40 mg dexamethasone given over 3 days for suspected ITP. The patient's platelet count normalized to 169,000 μL by day 4 of hospitalization, and he was discharged home in stable condition.

## DISCUSSION

Primary ITP is an autoimmune disorder characterized by isolated thrombocytopenia in the absence of other causes. Secondary ITP is mediated by other precipitating conditions such as rheumatologic disorders, lymphoproliferative diseases, malignancy, or infection.^3^ Viruses such as Epstein-Barr virus, HIV, and hepatitis C virus are commonly reported causes of ITP.^4^ According to current (2019) guidelines from the American Society of Hematology, first-line treatments for ITP are 4 doses of 40 mg dexamethasone and 2 doses of 1 mg/kg IVIG, with an alternative of rho (D) immunoglobulin.^5^ Suspected causes of thrombocytopenia in patients with COVID-19 include impaired bone marrow hematopoiesis, secondary hemophagocytic lymphohistiocytosis, sepsis-induced coagulopathy, and increased platelet destruction.^6^ Viral antigen-antibody complexes in response to viral infections result in platelet clearance by spleen macrophages.^7^ Immune-mediated destruction of platelets by ITP may be an additional etiologic consideration of thrombocytopenia in this patient population.^8^

SARS-CoV-2 infection appears to cause a profound immune response in patients. We suspect that our patients developed secondary ITP from COVID-19, as they had no known underlying autoimmune disorders. No other likely causes of thrombocytopenia were found after thorough investigation. Disseminated intravascular coagulation and sepsis-induced coagulopathy were unlikely, as both patients had clinically improved when their platelet counts declined. Additionally, coagulation studies were normal, and no evidence of active bleeding suggested disseminated intravascular coagulation or sepsis-induced coagulopathy.^9^ Thrombotic thrombocytopenic purpura was unlikely because neither patient had any observable or reported neurologic phenomenon, their renal functions were preserved, and they had no evidence of microangiopathic hemolytic anemia.^10^ Drug-induced thrombocytopenia was excluded after review of home medications.

Data on SARS-CoV-2–induced ITP are limited; however, a systematic review reported a small number of similar cases.^11^ ITP in this patient population may be more prevalent than currently documented.

## CONCLUSION

ITP is a diagnosis of exclusion. With the extraordinary recovery of the patients’ platelet counts after IVIG and steroid administration consistent with the treatment of ITP, a reasonable assumption is that these patients had autoimmune platelet destruction induced by SARS-CoV-2 infection. Clinicians who have ruled out other causes of thrombocytopenia in patients diagnosed with COVID-19 may reasonably consider guideline-directed treatment of ITP.

## Notes

*This article meets the Accreditation Council for Graduate Medical Education and the American Board of Medical Specialties Maintenance of Certification competencies for Patient Care and Medical Knowledge.*
